# Modulation of miR-26a-5p and miR-15b-5p Exosomal Expression Associated with Clopidogrel-Induced Hepatotoxicity in HepG2 Cells

**DOI:** 10.3389/fphar.2017.00906

**Published:** 2017-12-12

**Authors:** Renata C. Costa de Freitas, Raul H. Bortolin, Mariana B. Lopes, Letícia Tamborlin, Letícia Meneguello, Vivian N. Silbiger, Rosario D. C. Hirata, Mário H. Hirata, Augusto D. Luchessi, André D. Luchessi

**Affiliations:** ^1^Department of Clinical and Toxicological Analysis, Federal University of Rio Grande do Norte, Natal, Brazil; ^2^Laboratory of Biotechnology, School of Applied Sciences, University of Campinas, Limeira, Brazil; ^3^Post graduation in Biological Science, Institute of Biosciences, São Paulo State University (UNESP), Rio Claro, Brazil; ^4^Department of Clinical and Toxicological Analyses, School of Pharmaceutical Sciences, University of São Paulo, São Paulo, Brazil

**Keywords:** clopidogrel, microRNAs, hepatotoxicity, HepG2 cell line

## Abstract

Clopidogrel is an essential antiplatelet drug used to prevent thrombosis complications associated with atherosclerosis. However, hepatotoxicity is a potential adverse effect related to clopidogrel therapy. Exosome-derived miRNAs may be useful for improved monitoring of drug response and hepatotoxicity risk. In the present study, the expression of several exosomal miRNAs (miR-26a-5p, miR-145-5p, miR-15b-5p, and miR-4701-3p) and cell-derived mRNA targets (*PLOD2, SENP5, EIF4G2, HMGA2, STRADB*, and *TLK1*) were evaluated in HepG2 cells treated with clopidogrel (6.25, 12.5, 25, 50, and 100 μM) for 24 and 48 h. Then, clopidogrel cytotoxicity was evaluated by analyzing DNA fragmentation and the cell cycle profile using flow cytometry. Differential expression of exosome-derived miRNAs and cell-derived mRNAs was analyzed by RT-qPCR. Exposure of HepG2 cells to high concentrations of clopidogrel (50 and 100 μM) for 24 h caused significant DNA fragmentation (17.6 and 44.4%, respectively; *p* < 0.05) and 48 h (26.8 and 48.9%, respectively; *p* < 0.05), indicating cellular toxicity. Upregulation of miR-26a-5p and downregulation of miR-15b-5p was observed in cells exposed to 100 μM clopidogrel for 24 and 48 h. The miR-26a-5p target mRNAs *HMGA2, EIF4G2, STRADB*, and *SENP5* were downregulated in HepG2 cells following exposure to cytotoxic concentrations of clopidogrel (50 and 100 μM) for 24 h, and *HMGA2* levels remained low after 48 h of treatment. *TLK1*, a target of miR-15b-5p, was downregulated by 50 and 100 μM clopidogrel at 24 h. In conclusion, our results suggest that exposure to high concentrations of clopidogrel modulates the expression of exosomal miR-26a-5p and miR-15b-5p and their target mRNAs in HepG2 cells. Dysregulation of these miRNAs maybe modulate the regulatory pathways involved in clopidogrel-induced liver injury.

## Introduction

Clopidogrel is an antiplatelet therapy currently used for the prevention and treatment of thromboembolic complications and for delaying the progression of atherosclerosis (Fintel, 2012), a silent chronic vascular pathology that is a major cause of cardiovascular ischemic events (Badimon and Vilahur, 2014).

Clopidogrel is a pro-drug that is activated in the liver by several cytochrome P450 enzymes (CYPs) to the active metabolite, 2-oxo-clopidogrel, which inhibits platelet aggregation by binding irreversibly to purinergic receptor P2Y, G-protein coupled, 12 (P2Y12 receptor) (Sangkuhl et al., 2010; Fitzgerald and Fitzgerald, 2013; Yang et al., 2015). It can also be hydrolyzed, independent of CYP activity, by hepatic esterases, mainly carboxylesterase 1 (CES1), which generates the inactive carboxylic acid metabolite (CAM) (Beitelshees et al., 2015). Paraoxonase 1 (PON1) is involved in the biotransformation of 2-oxo-clopidogrel into a thiol active metabolite, which has been reported to be toxic (Bouman et al., 2011). The patient response to clopidogrel is highly variable, sometimes resulting in an increased risk of thrombotic events and adverse effects, which are important clinical problems in cardiovascular medicine (Beitelshees et al., 2015; Yang et al., 2015).

The most common adverse effects of clopidogrel treatment include gastrointestinal disorders, bleeding, thrombotic thrombocytopenic purpura, neutropenia, and aplastic anemia (Ochoa-Cortes et al., 2014; Kapila et al., 2015). Hepatotoxicity is a rare but potentially serious adverse effect related to clopidogrel treatment that can lead to fulminant hepatitis (Goyal et al., 2009; Monteiro et al., 2011; Zahno et al., 2013). However, the mechanisms of clopidogrel-associated hepatotoxicity have not been fully elucidated.

Advanced molecular analyses, particularly those used to measure gene expression, are useful for evaluating the changes related to organ dysfunction caused by adverse drug reactions, as characterized by the DNA damage that occurs during apoptosis (Vickers et al., 2017). In this way, the expression levels of various miRNAs have been proposed as novel biomarkers of drug-induced hepatotoxicity (Fontana, 2014; Weiler et al., 2015; Hayes and Chayama, 2016).

miRNAs are short, non-coding RNAs of 18–25 nucleotides that are partially complementary to target mRNAs. miRNAs play a crucial role in the post-transcriptional regulation of gene expression, mostly through gene silencing, as miRNA binding suppresses the translation of target mRNAs and/or promotes their degradation (Stroynowska-Czerwinska et al., 2014; Hayes and Chayama, 2016).

miRNAs are found in both intracellular and extracellular environments (circulating miRNAs). They are also detected in exosomes, which are 40–100 nm vesicles that are released from many types of cells into the extracellular space and are involved in cell-cell communication (Huang et al., 2013; Zhang et al., 2015). Exosomes also play an important role in the early release of miRNAs during drug-induced liver injury (Bala et al., 2012; McGill and Jaeschke, 2015).

Some studies have evaluated the role of miRNAs in the clopidogrel response. One study showed that platelet expression of miRNAs, such as miR-26a, was associated with clopidogrel resistance in patients who underwent coronary stenting (Chen et al., 2016). Platelet miRNAs were also proposed as potential predictors of reduced response to clopidogrel in acute coronary syndrome (Sunderland et al., 2017).

We previously used bioinformatics tools to investigate the association of several miRNAs and target mRNAs with the response to clopidogrel. We showed that five miRNAs, miR-145-5p, miR-26a-5p, miR-107, miR-15b-5p, and miR-4701-3p, were shown to influence platelet reactivity, clopidogrel response, and drug-induced toxicity, particularly hepatotoxicity (Freitas et al., 2016). This integrated analysis also showed that *SENP5, EIF4G2, HMGA2, STRADB*, and *TLK1*, which are potential targets of miR-26a and miR-15b, are associated with clopidogrel-induced hepatotoxicity (Freitas et al., 2016). *PLOD2* was also described as a potential target of miR-26a in a model of bladder cancer (Miyamoto et al., 2016).

In this study, we investigated the effect of cytotoxic concentrations of clopidogrel on the expression of miR-145, miR-26a, miR-4701, and miR-15b in exosomes and their target mRNAs in HepG2 cells.

## Materials and methods

### Cells and culture

HepG2 cells were obtained from the Rio de Janeiro Cell Bank (Rio de Janeiro, Brazil) and maintained in RPMI-1640 medium (pH 7.4) supplemented with L-glutamine (2 mM, penicillin (100 U/mL), streptomycin (100 μg/mL), and 5% exosome-depleted fetal bovine serum. The cells were grown in cell culture flasks at 37°C in a humidified atmosphere containing 5% CO_2_ to 80–90% confluence.

### Treatment of HepG2 cells with clopidogrel

For flow cytometry analysis, HepG2 cells were seeded in 24-well plates (1.5 × 10^5^ cells/well) and maintained in culture medium for 24 h. Then, the cells were treated with 0.0 (vehicle), 6.25, 12.5, 25, 50, and 100 μM clopidogrel (Sigma-Aldrich, St. Louis, MO, USA) dissolved in dimethylsulfoxide (DMSO) at a final concentration of 0.1% for 24 and 48 h.

For the miRNA and mRNA expression analyses, HepG2 cells were seeded in 150 cm^2^ plates (9 × 10^6^ cells/plate) and maintained in culture medium for 24 h. Then, the cells were treated for 24 and 48 h with 0.0 (vehicle), 6.25, 12.5, 25, 50, and 100 μM clopidogrel dissolved in DMSO at final concentration of 0.1%.

### Analysis of clopidogrel cytotoxicity by flow cytometry

DNA fragmentation and the cell cycle were analyzed by flow cytometry. HepG2 cells exposed to clopidogrel were collected by trypsinization, centrifuged at 200 × g for 5 min at room temperature (~25°C) and washed with 500 μL of PBS. Cell pellets were fixed with 500 μL of 70% (v/v) cold ethanol. Fixed cells were washed with PBS and then resuspended in 500 μL of propidium iodide (PI) solution (20 μg/mL of PI, 0.1% Triton X-100, and 10 μg/mL DNAse free RNAse in PBS) and incubated for 30 min in the dark.

Flow cytometry analysis was carried out using a BD Accuri™ C6 Plus Cytometer (BD Bioscience, San Jose, CA, USA). Ten-thousand events were evaluated in each sample test. Data were collected from three independent experiments, performed in triplicate. Cells displaying hypodiploid DNA content (sub-G1) were marked as apoptotic.

Cell supernatants were used to measure the levels of alanine transaminase (ALT) and aspartate transaminase (AST), two markers of liver injury, by colorimetric-enzymatic methods using a biochemical analyzer (BIO-2000 IL; Bioplus Products for Laboratories, Sao Paulo, Brazil).

### RNA extraction from exosomes and HepG2 cells

Exosomes were isolated from the supernatant of HepG2 cells treated with clopidogrel (12.5, 25, 50, and 100 μM) or vehicle (control) using the exoRNeasy Serum/Plasma Maxi kit (Qiagen, Hilden, Germany; Cat. Number: 77064), according to the manufacturer's recommendations. Briefly, pre-filtered supernatants from treated cells were mixed 1:1 with binding buffer and added to an exoEasy membrane affinity column to allow the exomes bind to the membranes. The columns were centrifuged at 500 × g for 1 min at room temperature (~25°C), and washed with washing buffer to remove non-specifically retained materials.

The exosomes were lysed by adding QIAzol (Qiagen) to the columns, and then the lysates were collected by centrifugation (Enderle et al., 2015). The *Caenorhabditis elegans* miR-39 (cel-miR-39), which is the Spike-In Control contained in the miRNeasy Serum/Plasma Kit (Qiagen; Cat. Number: 219610) was added to monitor RNA recovery and reverse transcription efficiency. RNA was quantified and purity was assessed by spectrophotometry using a Nanodrop ND-1000 (Thermo Scientific, Wilmington, DE, USA).

Total RNA was extracted from clopidogrel-treated HepG2 cells using TRIzol reagent (Invitrogen, Carlsbad, CA, USA) according to the manufacturer's protocol. RNA was quantified and purity was assessed by spectrophotometry using a Nanodrop ND-1000.

### Exosomal miRNA expression by RT-qPCR

The cDNA of the miRNAs was synthesized with the miScript II RT Kit (Qiagen; Cat. Number: 218161) according to the manufacturer's protocol using a Veriti™ 96-Well Thermal Cycler (Applied Biosystems, Carlsbad, CA, USA).

RT-qPCR analysis was performed with pre-validated miScript® Primer Assays (Qiagen; Cat. Number: 218300) for the following miRNAs: Hs_miR-26a_2 (MIMAT0000082), Hs_miR-145_1 (MIMAT0000437), Hs_miR-15b_2 (MIMAT0000417), Hs_miR-4701-3p_1 (MIMAT0019799), and Hs_miR-107_2 (MIMAT0000104) using the miScript SYBR Green PCR Kit (Qiagen; Cat. Number: 218073). These miRNAs were select based on the results of our previous *in silico* study (Freitas et al., 2016).

RT-qPCR assays were carried out in 96-well plates using a 7500 Fast Real-Time PCR System (Applied Biosystems). The amplification program consisted of 40 cycles of 94°C for 15 s, 55°C for 30 s, and 70°C for 30 s. Data were collected from three independent experiments performed in duplicate. miRNAs with Ct-values <37 were excluded from the statistical analysis.

miRNA expression was normalized to relative quantities (NRQs) in three steps, as previously described (Marabita et al., 2016): (i) the miRNA target Ct-values were scaled with cel-miR-39 (formula: RQ = miRNA target Ct – cel-miR-39 Ct); (ii) the geometric RQ means of all expressed miRNAs per sample were obtained to calculate the normalization factor (NF); and (iii) the NRQ-values were calculated using the formula: NRQ = miRNA target RQ/NF. The results are shown as the fold change (FC), which was calculated using the NRQ-values of clopidogrel-treated cells (6.25, 12.5, 25, 50, and 100 μM) divided by NRQs-values of control cells (vehicle only). The NRQ-values obtained in the experiments are shown in Supplementary Table 2.

### RT-qPCR analysis of mRNA expression in HepG2 cells

The cDNA was synthesized with 2 μg of total RNA using the SuperScript® III First-Strand Synthesis System (Invitrogen), according to the manufacturer's protocol using a Veriti™ 96-Well Thermocycler (Applied Biosystems).

RT-qPCR was carried out in 96-well plates using primers specific for *PLOD2, SENP5, EIF4G2, HMGA2, STRADB, TLK1*, and *GAPDH* (Supplementary Table 1) and the QuantiTect SYBR Green PCR Kit (Qiagen; Cat. Number: 204145). The qPCR assays were carried in a 7500 Fast Real-Time PCR System (Applied Biosystems). The amplification program consisted of 40 cycles of 94°C for 15 s, 55°C for 30 s, and 70°C for 30 s. Data were collected from three independent experiments performed in duplicate. mRNAs with Ct-values <37 were excluded from the statistical analysis.

*GAPDH, 18S rDNA*, and *ACTB* were used as reference genes. According to the NormFinder algorithm, *GAPDH* was the most stable gene under our experimental conditions, thus it was used as the endogenous control. Relative expression was calculated by the 2^−ΔΔCt^ method (Livak and Schmittgen, 2001), using vehicle (0 μM clopidogrel) treated cells as the control. The delta Ct-values obtained in the experiments are shown in Supplementary Table 3.

Previously published studies and data available at the National Center for Biotechnology Information (NCBI PubMed; https://www.ncbi.nlm.nih.gov/pubmed/) for indicated the regulatory pathway of miRNAs and their differently expressed mRNA targets were used.

### Statistical analysis

The statistical analysis was carried out using SPSS® 20 software (IBM, New York, NY, USA). DNA fragmentation data are shown as means and standard error of the mean (SEM), and miRNA and mRNA expression data are shown as the fold change between clopidogrel-treated (6.25, 12.5, 25, 50, and 100 μM) and control (vehicle only, 0 μM clopidogrel) cells. The relative expression of the miRNAs and mRNAs in clopidogrel-treated cells were compared to those in control cells (vehicle) by Kruskal–Wallis ANOVA, and pairwise comparisons were performed with the Mann–Whitney test. A *p* < 0.05 was considered statistically significant.

## Results

### Toxic effects of clopidogrel in HepG2 cells

**Figure 1:**
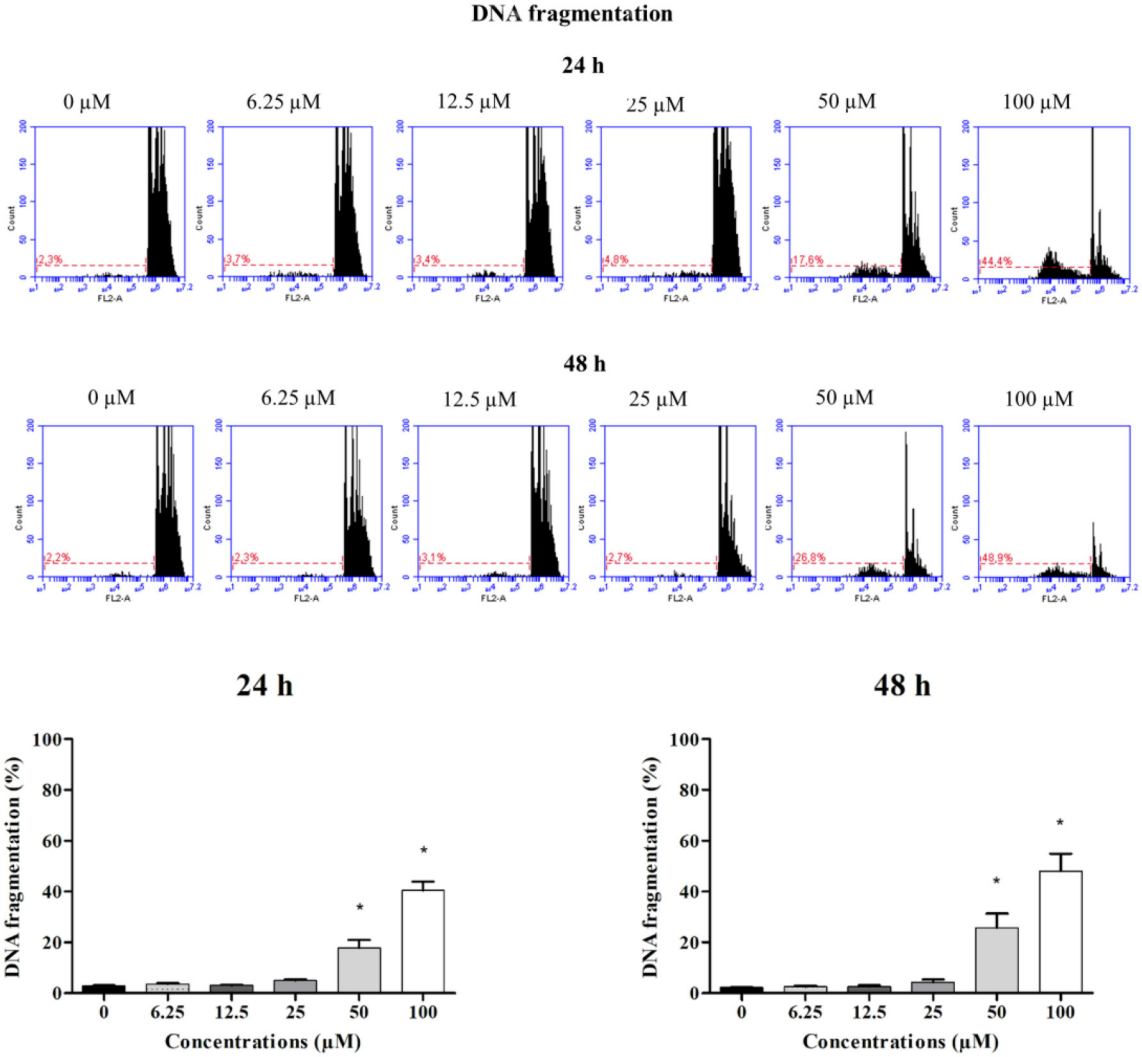
DNA fragmentation in HepG2 cells treated with clopidogrel. Representative histograms and percentage of cells with fragmented DNA after treatment with clopidogrel (6.25, 12.5, 25, 50, and 100 μM) for 24 and 48 h. Data are shown as mean ± SEM of three independent experiments performed in triplicate and compared by Kruskal–Wallis ANOVA and pairwise comparisons by Mann–Whitney test. ^*^p < 0.05 compared to control (vehicle, 0 μM clopidogrel).

**Figure 2:**
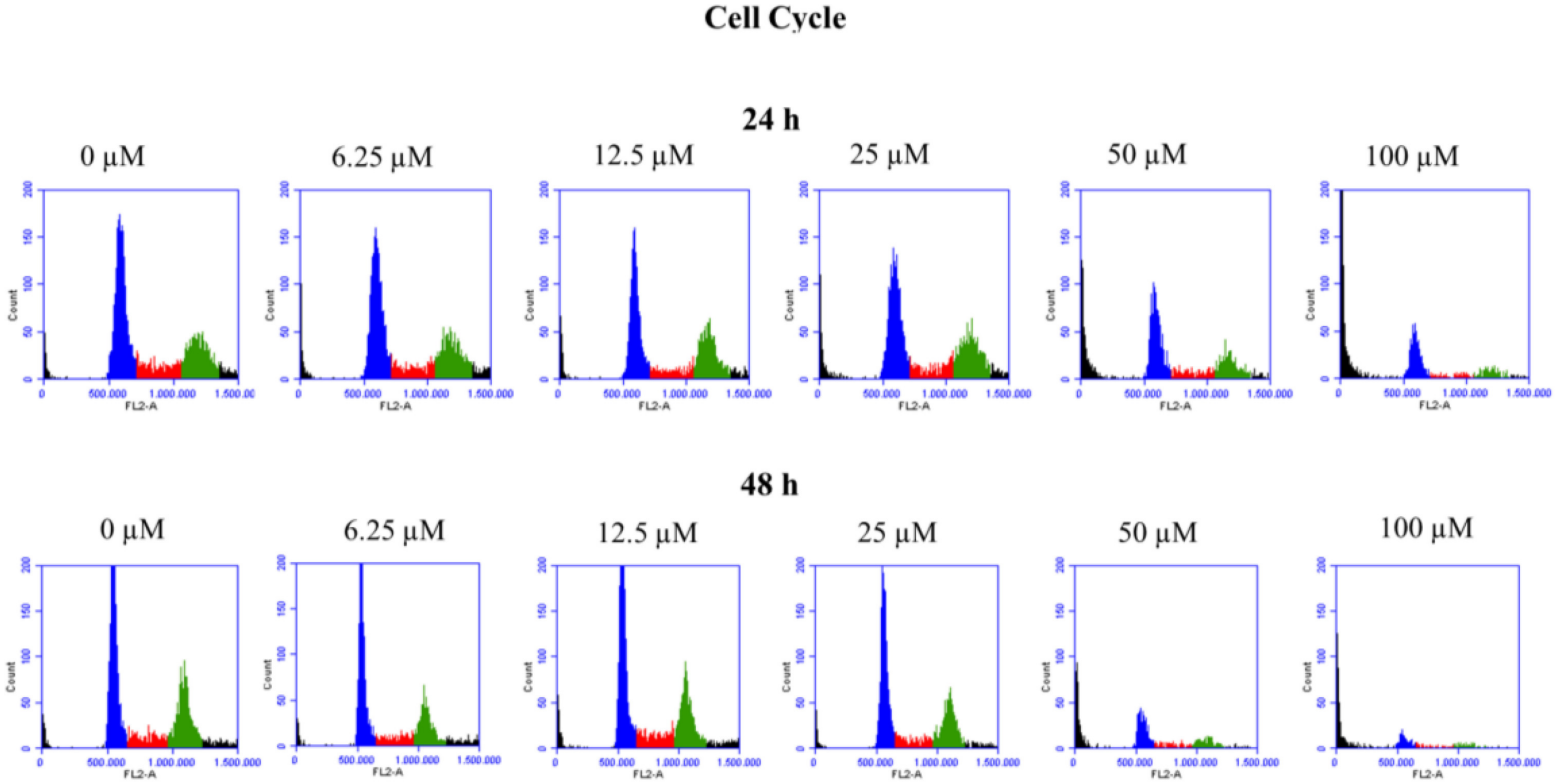
Cell cycle profile of HepG2 cells treated with clopidogrel. Representative histograms of cells treated with clopidogrel (6.25, 12.5, 25, 50, and 100 μM) for 24 and 48 h. Blue: cells in the G0/G1 phase; Red: cells in the S phase; Green: cells in the G2/M phase. Data from 3 independent experiments performed in triplicate.

To verify the toxic effects of clopidogrel in a human liver cell line, HepG2 cells were treated with different concentrations of clopidogrel (6.25, 12.5, 25, 50, and 100 μM) for 24 and 48 h. Flow cytometry analysis showed that cells treated with 50 and 100 μM clopidogrel showed a higher percentage of cells with fragmented DNA than control cells at 24 h (17.6 and 44.4%, respectively; *p* < 0.05) and 48 h (26.8 and 48.9%, respectively; *p* < 0.05; Figure 1). In addition, the cell cycle profile of these cells showed general damage following clopidogrel treatment (Figure 2). These results suggest that clopidogrel, at 50 and 100 μM, is potentially toxic to HepG2 cells. In contrast, cells treated with 6.25, 12.5, and 25 μM clopidogrel did not show significant DNA fragmentation at 24 h (3.7, 3.4, and 4.8%, respectively) or 48 h (2.3, 3.1, and 2.7%, respectively) when compared to the levels in control cells (2.3 and 2.2% at 24 and 48 h, respectively; *p* > 0.05). These lower concentrations of clopidogrel also did not affect the cell cycle profile, indicating that these are non-toxic concentrations for HepG2 cells.

Analysis of AST and ALT activities in HepG2 cell supernatants showed similar values for clopidogrel-treated (12.5, 25, 50, and 100 μM) and control cells at 24 and 48 h (*p* > 0.05; Supplementary Figure 1).

### Expression of exosome-derived miRNAs

**Figure 3:**
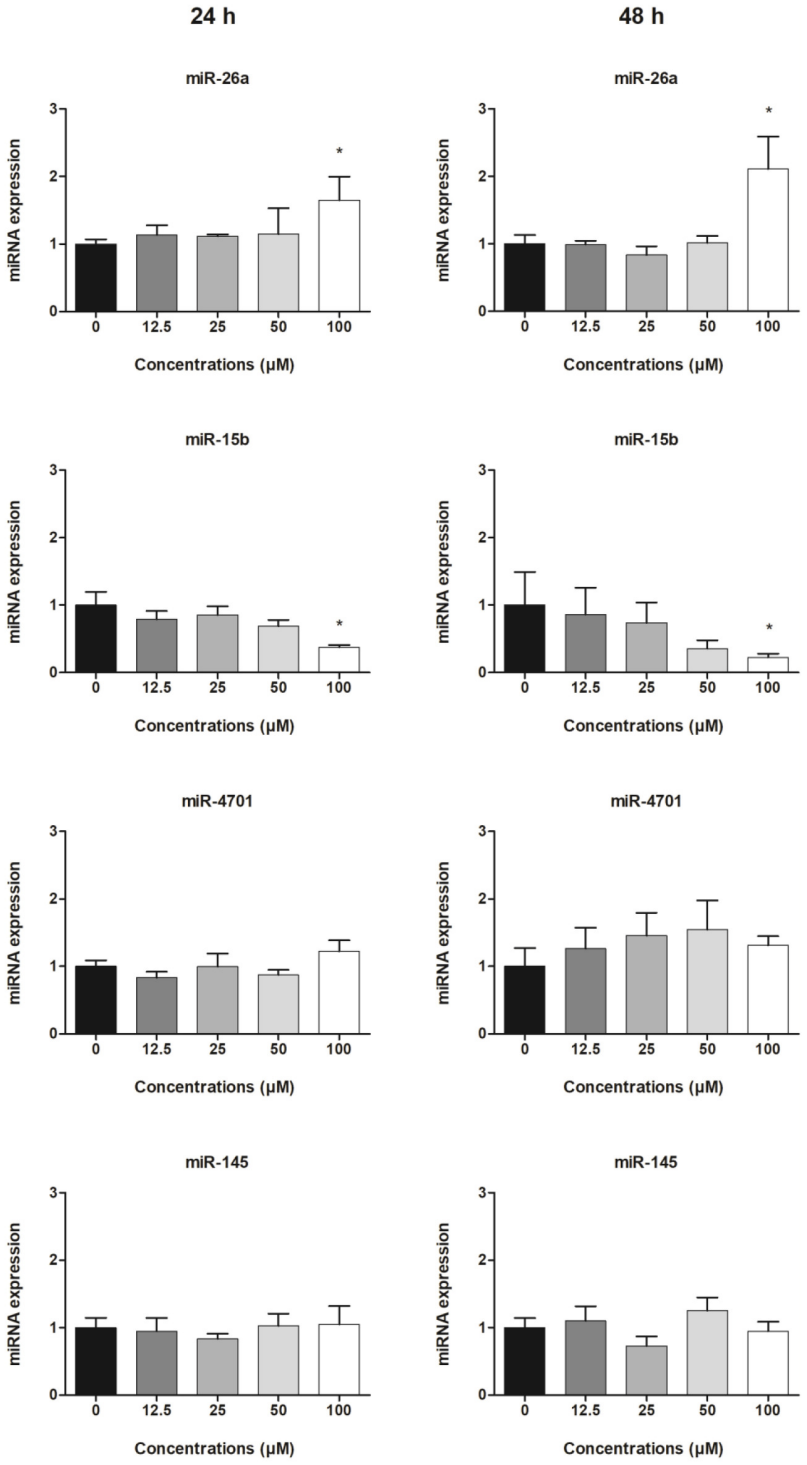
Expression of exosomal miRNAs derived from HepG2 cells treated with clopidogrel. Cells were treated with clopidogrel (6.25, 12.5, 25, 50, and 100 μM) for 24 h and 48 h. Data are shown as fold change of normalized relative quantities (NRQ) of clopidogrel-treated cells divided by control cells (vehicle, 0 μM clopidogrel), and analyzed by Kruskal–Wallis ANOVA and pairwise comparisons by Mann–Whitney test. ^*^p < 0.05 compared to control. Data from 3 independent experiments were performed in duplicate.

The relative expression levels (fold changes) of exosome-derived miRNAs in the supernatants of HepG2 cells treated with different concentrations of clopidogrel are shown in Figure 3. After 24 and 48 h of exposure to 100 μM clopidogrel, exosome miR-26a was upregulated (two-fold) and miR-15b was downregulated (four-fold) when compared to the levels in the control (*p* < 0.05). In contrast, the expression of miR-145 and miR-4701 in HepG2-derived exosomes was not significantly influenced by clopidogrel treatment (*p* > 0.05). miR-107 was not further analyzed due to having a Ct-value above 37.

### Expression of HepG2 cell-derived mRNAs

**Figure 4:**
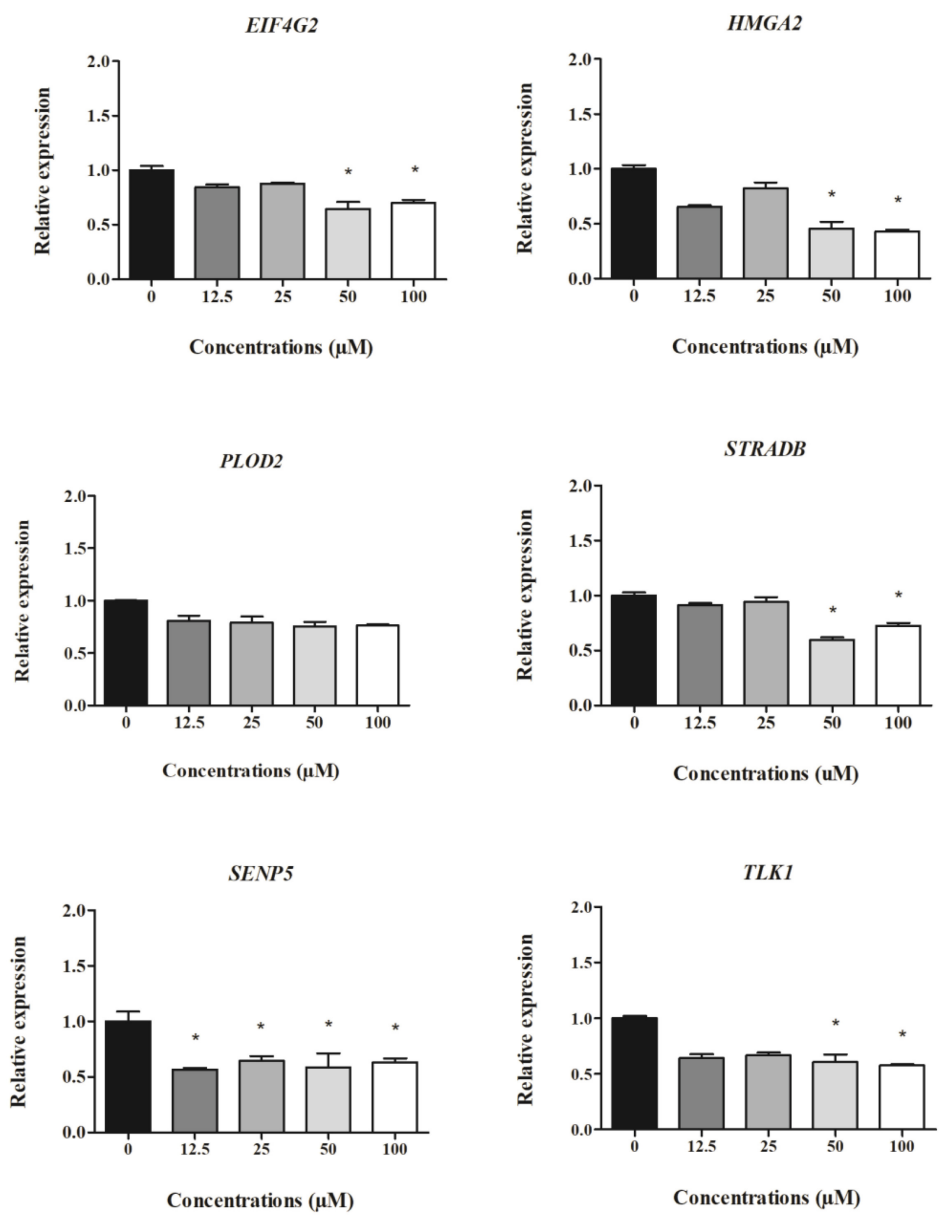
Expression of cell-derived mRNAs in HepG2 cells after 24 h treatment with clopidogrel (12.5, 25, 50, and 100 μM). Data are shown as fold change (2^−ΔΔCt^) of clopidogrel-treated cells, using the vehicle (0 μM clopidogrel), as control and compared by Kruskal–Wallis ANOVA and pairwise comparisons by Mann–Whitney test. ^*^p < 0.05 compared to control. Data from three independent experiments performed in duplicate.

**Figure 5:**
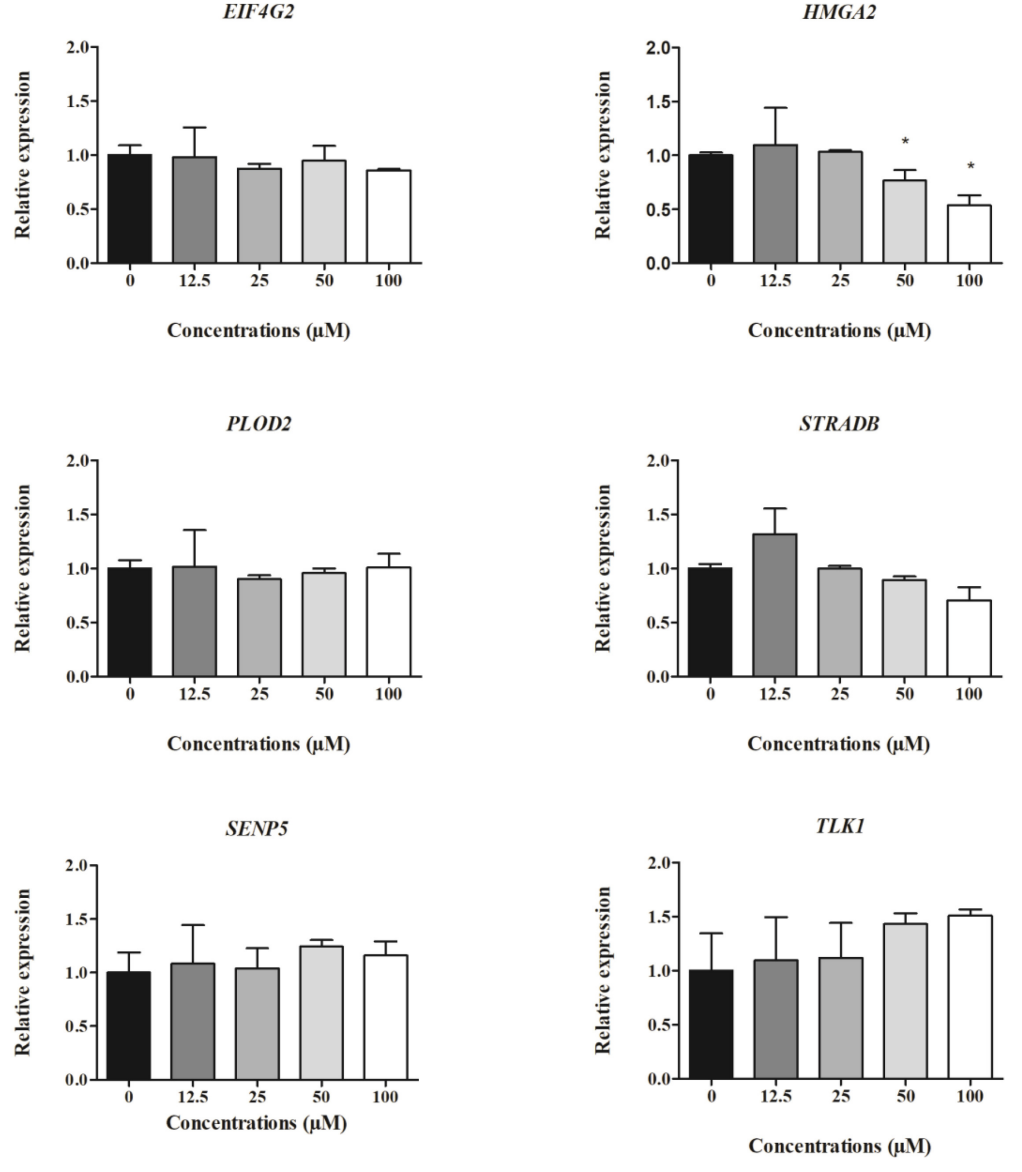
Expression of cell-derived mRNAs in HepG2 cells after 48 h treatment with clopidogrel (12.5, 25, 50, and 100 μM). Data are shown as fold change (2^−ΔΔCt^) of clopidogrel-treated cells, using the vehicle (0 μM clopidogrel), as control and compared by Kruskal–Wallis ANOVA and pairwise comparisons by Mann–Whitney test. ^*^p < 0.05 compared to control. Data from three independent experiments performed in duplicate.

The relative expression of cell-derived mRNAs in cells treated with different concentrations of clopidogrel after 24 and 48 h is shown in Figures 4, 5, respectively. *EIF4G2, HMGA2, STRADB, SENP5*, and *TLK1* levels in HepG2 cells were two-fold lower after 24 h of exposure to cytotoxic concentrations of clopidogrel (50 and 100 μM) than the levels in control cells (*p* < 0.05; Figure 4). *SENP5* mRNA levels were also reduced in cells treated with non-cytotoxic concentrations of clopidogrel (12.5 and 25 μM) for 24 h (*p* < 0.05) when compared to the levels in control cells. Conversely, *PLOD2* mRNA expression did not vary in cells treated with any concentration of clopidogrel for 24 h.

After 48 h of clopidogrel treatment, *HMGA2* expression remained downregulated in cells treated with cytotoxic concentrations of clopidogrel (50 and 100 μM) when compared to the expression in the control (*p* < 0.05; Figure 5). However, the mRNA levels of *EIF4A2, PLOD2, STRADB, SENP5*, and *TLK1* were not altered by 48 h of treatment with clopidogrel (*p* > 0.05).

The regulatory pathways associated with these differentially expressed exosome-derived miRNAs and their target mRNAs are shown in Table 1. The regulatory pathways for miR-26a were liver regeneration, cell cycle control, and apoptosis. The targets of miR-26a, *HMGA2, EIF4G2, STRADB*, and *SENP5*, are involved in cell growth and development, cell cycle control, DNA damage, and apoptosis. miR-15b is involved in cell survival and cell cycle control, and its target, *TLK1*, is associated with cell cycle control.

**Table 1:** miRNAs-mRNAs regulatory pathways.

**miRNA**	**Change**	**target mRNA**	**Change**	**Regulatory pathway**	**References**
miR-26a-5p	Upregulation			Liver regeneration, cell cycle control, apoptosis	Lizarraga et al., 2012; Zhou et al., 2012; Zhu et al., 2012; Freitas et al., 2016
		*HMGA2*	Downregulation	Cell growth and development	Sun et al., 2013; Zhou et al., 2014
		*EIF4G2*	Downregulation	DNA damage and apoptosis	Badura et al., 2012
		*STRADB*	Downregulation	Cell cycle control	Zhong et al., 2013
		*SENP5*	Downregulation	Cell growth and apoptosis	Di Bacco et al., 2006; Wang and Zhang, 2014
miR-15b-5p	Downregulation			Cell survival and cell cycle control	van Rooij et al., 2012
		*TLK1*	Downregulation	Cell cycle control	Kim et al., 2016; Timiri Shanmugam et al., 2016

*Regulatory pathways were obtained from data published in National Center for Biotechnology Information (NCBI PubMed; https://www.ncbi.nlm.nih.gov/pubmed/)*.

## Discussion

In this study, the toxic effects of clopidogrel treatment on exosomal miRNAs and their cellular target mRNAs were evaluated in HepG2 cells. Exposure to high concentrations of clopidogrel (50 and 100 μM) led to fragmented DNA in a large number of cells as well as general damage as observed by the altered cell cycle profile of clopidogrel-treated HepG2 cells, which are suggestive of clopidogrel cytotoxicity. Previous studies suggested that the levels of clopidogrel in the liver of patients treated with a maintenance dose of 75 mg per day may reach 10 μM (Caplain et al., 1999; Zahno et al., 2010, 2013). Likewise, 50 and 100 μM clopidogrel are equivalent to the loading standard dose (300 mg) and high dose (600 mg) prescribed for percutaneous coronary intervention (angioplasty) protocols.

In a previous study, 10 and 100 μM clopidogrel were shown to be toxic to primary human hepatocytes when combined with rifampicin (an inducer of CYP3A4) and to HepG2 cells overexpressing CYP3A4 (Zahno et al., 2013). In addition, high concentrations of clopidogrel (100 and 300 μM) were significantly toxic to primary rat hepatocytes and HepG2 cells co-incubated with recombinant CYP2B6 and CYP2C19 (Zhai et al., 2016). These effects have been attributed to 2-oxo-clopidogrel, a bioactive metabolite, produced by CYP enzymes (Maffrand, 2012; Zhai et al., 2016). In contrast, it has been shown that high concentration (100 μM) of clopidogrel and its major inactive metabolite (CAM) are not toxic to wild-type HepG2 cells (Zahno et al., 2013). The lack of clopidogrel toxic effect on HepG2 cells reported previously may be due to differences in the methodology used for cytotoxicity assessment compared to our study.

In HepG2 cells, CYPs have low activity, particularly CYP1A2, CYP2B6, and CYP3A4 (Gerets et al., 2012; O'Brien, 2014). The low expression levels of these CYP enzymes combined with the lack of CAM toxicity in HepG2 cells suggest that clopidogrel (pro-drug) itself can cause hepatocellular lesions. This is particularly important in patients with low CYP activity due to genetic variations (e.g., CYP2C19^*^2, poor metabolizers, who are exposed to high concentrations of clopidogrel than the CYP2C19^*^1, extensive metabolizers) or drug-drug interactions for both the standard- and high-dose regimens.

In this study, high doses of clopidogrel altered the exosomal-derived miRNAs miR-26a and miR-15b. These miRNAs have been shown to be associated with liver regeneration, cell cycle control, cell growth, survival, development, DNA damage, and apoptosis.

In accordance with our previous *in silico* study, we observed upregulation of exosomal miR-26a levels following exposure to a cytotoxic concentration (100 μM) of clopidogrel, which suggested that miR-26a upregulation may play an important role in the clopidogrel response and hepatotoxicity (Freitas et al., 2016). Likewise, increased miR-26a expression was also observed in a toxicological study of HepG2 cells using benzo[*a*]pyrene (BaP), a polycyclic aromatic hydrocarbon, as the genotoxin/carcinogen. It has been suggested that miR-26a modulates important mRNA networks involved in toxicity, in response to BaP treatment (Lizarraga et al., 2012). Overexpression of the miR-26 family also resulted in an accumulation of the G1 population of HepG2 cells, through inhibition of cell cycle progression (Zhu et al., 2012). Conversely, downregulation of miR-26a induced hepatocyte proliferation during liver regeneration in male C57BL/6J mice (Zhou et al., 2012).

In this study, the cell cycle profile of HepG2 cells treated with toxic concentrations (50 and 100 μM) of clopidogrel may be caused by a combination of apoptosis and the effect of miR-26 on cell cycle control.

In this study, the expression levels of the miR-26a mRNA targets *HMGA2, STRADB, EIF4G2*, and *SENP5* in HepG2 cells were significantly downregulated following exposure to a toxic concentration (100 μM) of clopidogrel. These genes are involved with general cellular processes, such as cell growth and development, cell cycle control, DNA damage, and apoptosis.

*HMGA2* encodes high mobility group at-hook 2, which is an architectural protein involved in various cellular processes, such as uncontrolled growth and development and alteration of chromatin architecture, that acts by modifying the interaction of transcriptional enhancers (Sun et al., 2013). A previous study showed that *HMGA2* mRNA levels were inversely correlated with miR-26a expression and that reduced levels of *HMGA2* inhibited the proliferation of gallbladder cancer cells (Zhou et al., 2014). Therefore, downregulation of *HMGA2* may be involved in the cytotoxic mechanism of clopidogrel in HepG2 cells, by affecting cell growth and development.

*STRADB* encodes STE20-related kinase adaptor beta, which is involved in the induction of cell cycle arrest by controlling the relocation of serine/threonine protein kinase 11 (STK11 or LKB1), from the nucleus to the cytoplasm (Zhong et al., 2013). It is likely that reduced expression of *STRADB* is associated with increased cell cycle length and consequent slowing down of the cell cycle, especially at G1 phase. This hypothesis is supported by the DNA fragmentation and cell cycle data collected using HepG2 cells exposed to toxic concentrations of clopidogrel.

Eukaryotic translation initiation factor 4-gamma 2 is encoded by *EIF4G2*. It has been shown that silencing of *EIF4G2* increases the DNA damage induced by ionizing radiation in breast cancer cells (Badura et al., 2012). Moreover, reduced levels of *EIF4G2* have been suggested to be involved in apoptosis (Signoretti et al., 2010). It is likely that downregulation of *EIF4G2* led to the increased DNA fragmentation and apoptosis observed in response to clopidogrel. This cytotoxic mechanism may be associated with an accumulation of reactive oxygen species and mitochondrial damage in the presence of the clopidogrel (Zahno et al., 2013; Zhai et al., 2016).

*SENP5* encodes sentrin-specific protease 5, a C-terminal hydrolase that activates small ubiquitin-like SUMO proteins, which are involved in several biologic processes, including cell division and proliferation (Di Bacco et al., 2006). Inhibition of *SENP5* was shown to suppress cell growth and induce apoptosis (Wang and Zhang, 2014). In this study, all tested concentrations of clopidogrel downregulated *SENP5* mRNA levels; therefore, other mechanisms, besides the miR-26a pathway, may be involved in the regulation of *SENP5* by clopidogrel.

*PLOD2* is also a target mRNA of miR-26a. However, in this study, clopidogrel did not alter *PLOD2* expression in HepG2 cells, which suggests that *PLOD2* is not involved in the cytotoxic mechanism of clopidogrel.

Exosomal miR-15b was regulated by toxic concentrations (100 μM) of clopidogrel after 48 h of treatment. Downregulation of miR-15b was also reported in TM4 cells treated with the cytotoxic xenobiotic nonylphenol for 24 h (Choi et al., 2011) and in cardiomyocytes derived from human pluripotent stem cells exposed to doxorubicin (Holmgren et al., 2016). These results suggest that suppression of miR-15b may be involved in the cytotoxic mechanism of clopidogrel and other drugs. Although the exact functions of miR-15b are still being explored, miR-15b has been implicated in cell survival and cell cycle regulation under different clinical conditions, such as cancer and cardiac diseases (van Rooij et al., 2012). The role of miR-15b in liver cells has not been fully elucidated, and more studies are needed to investigate its regulatory effects in the cell survival and cell cycle control pathways.

We previously suggested that miR-15b may be a regulator of *TLK1* (Freitas et al., 2016). In this study, miR-15b and *TLK1* levels in HepG2 cells were downregulated by cytotoxic levels of clopidogrel. This result suggests that other molecules are involved in the modulation of *TLK1* expression following exposure to clopidogrel. *TLK1* encodes tousled-like kinase 1, a nuclear serine/threonine kinase that regulates chromatin assembly. *TLK1* repression resulted in delayed S-phase progression in luminal breast cancers, suggesting its important role in cell cycle regulation (Kim et al., 2016). TLK1 was also involved in DNA damage-induced checkpoint arrest and suppressed cytotoxicity in salivary gland cells (Timiri Shanmugam et al., 2016). Therefore, *TLK1* downregulation may be also involved in clopidogrel-induced hepatotoxicity, by controlling the cell cycle.

In this study, exosomal expression of miR-145 and miR-4701 was not affected by clopidogrel. miR-145 has been associated with suppression of cell proliferation, invasion, and migration and induction of apoptosis in melanoma cells (Liu et al., 2017; Moon et al., 2017).

The mechanisms underlying clopidogrel-induced hepatotoxicity have not been fully elucidated. The general mechanisms of hepatotoxicity involve cellular accumulation of reactive oxygen species and opening of the mitochondrial permeability transition pore, leading to a release of cytochrome c into the cytoplasm, which is a key event in apoptosis (Zahno et al., 2013; Zhai et al., 2016), supporting our finding that clopidogrel-induced hepatotoxicity is directly related to apoptosis. Moreover, considering that this adverse effect is idiosyncratic (Kapila et al., 2015), understanding the regulatory mechanism will promote future studies attempting to validate the epigenetic mechanism involved in drug-induced hepatotoxicity.

In this study, high concentrations of clopidogrel led to dysregulated expression of exosomal miRNAs and target mRNAs in HepG2 cells. However, these effects were not related to changes in supernatant levels of AST and ALT, two markers of liver injury. It is likely that changes in exosomal miRNA expression are early events related to clopidogrel cell toxicity, and that dysregulation of miR-26a and miR-15b may be useful markers of drug-induced toxicity in clinical settings (Bronze-da-Rocha, 2014; Marrone et al., 2015; Yu et al., 2016).

It would be interesting to expand this analysis of cellular miRNA expression in clopidogrel-treated HepG2 cells to an investigation of the qualitative and quantitative distribution of miRNAs in source cells and their exosomes, which could provide more information regarding the contribution of circulating miRNAs for clinical evaluation of drug-induced liver injury.

In conclusion, toxic concentrations of clopidogrel modulate the expression of miR-26a and miR-15b and their mRNA targets *EIF4G2, HMGA2*, and *STRADB* in HepG2 cells, which regulate the cell cycle and apoptosis, mechanisms that maybe involved in clopidogrel-induced liver injury.

## Author contributions

AnDL and VS were responsible for the original concept of the study, as well as the study design; RF, RB, ML, LT, and LM performed the experimental study and laboratory analyses; AnDL, VS, AuDL, MH, RH, RB, and RF interpreted the results and performed the statistical analyses; RF and RB wrote the manuscript; AnDL, AuDL, and MH were responsible for financial support; AnDL, AuDL, RH, and MH critically revised the manuscript.

### Conflict of interest statement

The authors declare that the research was conducted in the absence of any commercial or financial relationships that could be construed as a potential conflict of interest.
