# Supplementary material for: Modulation of miR-26a-5p and miR-15b-5p Exosomal Expression Associated with Clopidogrel-Induced Hepatotoxicity in HepG2 Cells
Source: Front Pharmacol. 2017 Dec 12;8:906. doi: 10.3389/fphar.2017.00906 (PMC5733064; doi:10.3389/fphar.2017.00906)
Supplement: Supplementary file 3 [file Table3.docx]

Supplementary Material

Modulation of miR-26a-5p and miR-15b-5p exosomal expression associated with clopidogrel-induced hepatotoxicity in HepG2 cells

Renata Caroline Costa de Freitas, Raul Hernandes Bortolin, Mariana Borges Lopes, Letícia Tamborlin, Letícia Meneguello, Vivian Nogueira Silbiger, Rosario Dominguez Crespo Hirata, Mário Hiroyuki Hirata, Augusto Ducati Luchessi, André Ducati Luchessi^*^

* Corresponding author

André Ducati Luchessi

E-mail: [andre.luchessi@outlook.com](mailto:andre.luchessi@outlook.com)

# SUPPLEMENTARY TABLES

**Suppl. Table 3.** mRNAs expression as delta Ct values in HepG2 cells treated with clopidogrel for 24 and 48 h

| **mRNA** | **Clopidogrel concentration** | | | | |  |
| --- | --- | --- | --- | --- | --- | --- |
|  | **0µM** | **12.5µM** | **25µM** | **50µM** | **100µM** | ***p-value*** |
|  | **24 h Treatment** | | | | |  |
| *EIF4G2* | 0.110 ± 0.004 | 0.092 ± 0.002 | 0.095 ± 0.002 | 0.079 ± 0.006 | 0.079 ± 0.003 | *0.0066* |
| *HMGA2* | 0.026 ± 0.001 | 0.017 ± 0.000 | 0.022 ± 0.001 | 0.012 ± 0.001 | 0.011 ± 0.000 | *0.0073* |
| *PLOD2* | 0.002 ± 0.000 | 0.001 ± 0.000 | 0.001 ± 0.000 | 0.001± 0.000 | 0.001 ± 0.000 | *0.0616* |
| *SENP5* | 0.008 ± 0.000 | 0.005 ± 0.000 | 0.006 ± 0.000 | 0.005 ± 0.000 | 0.005 ± 0.000 | *0.0472* |
| *STRADB* | 0.009 ± 0.000 | 0.008 ± 0.000 | 0.009 ± 0.000 | 0.006 ± 0.000 | 0.007 ± 0.000 | *0.0019* |
| *TLK1* | 0.025 ± 0.000 | 0.018 ± 0.000 | 0.017 ± 0.000 | 0.016 ± 0.001 | 0.015 ± 0.000 | *0.0480* |
|  | **48 h Treatment** | | | | |  |
| *EIF4G2* | 0.081 ± 0.014 | 0.099 ± 0.022 | 0.071 ± 0.011 | 0.096 ± 0.011 | 0.084 ± 0.002 | *0.8613* |
| *HMGA2* | 0.017 ± 0.000 | 0.019 ± 0.006 | 0.018 ± 0.000 | 0.013 ± 0.001 | 0.009 ± 0.001 | *0.0412* |
| *PLOD2* | 0.001 ± 0.000 | 0.002 ± 0.000 | 0.001 ± 0.000 | 0.001± 0.000 | 0.001 ± 0.000 | *0.0531* |
| *SENP5* | 0.004 ± 0.000 | 0.005 ± 0.001 | 0.004 ± 0.000 | 0.006 ± 0.000 | 0.007 ± 0.000 | *0.4195* |
| *STRADB* | 0.005 ± 0.000 | 0.006 ± 0.001 | 0.005 ± 0.000 | 0.004 ± 0.000 | 0.003 ± 0.000 | *0.0730* |
| *TLK1* | 0.010 ± 0.003 | 0.009 ± 0.004 | 0.012 ± 0.003 | 0.015 ± 0.001 | 0.016 ± 0.000 | *0.6718* |

Delta Ct = Ct specific mRNA – Ct GAPDH. Data are shown as mean ± standard error and compared by Kruskal-Wallis.
