# Supplementary material for: Modulation of miR-26a-5p and miR-15b-5p Exosomal Expression Associated with Clopidogrel-Induced Hepatotoxicity in HepG2 Cells
Source: Front Pharmacol. 2017 Dec 12;8:906. doi: 10.3389/fphar.2017.00906 (PMC5733064; doi:10.3389/fphar.2017.00906)
Supplement: Supplementary file 4 [file Image1.pdf]

Supplementary Material

Modulation of miR-26a-5p and miR-15b-5p exosomal expression associated with clopidogrel-induced hepatotoxicity in HepG2 cells

Renata Caroline Costa de Freitas, Raul Hernandes Bortolin, Mariana Borges Lopes, Letícia Tamborlin, Letícia Meneguello, Vivian Nogueira Silbiger, Rosario Dominguez Crespo Hirata, Mário Hiroyuki Hirata, Augusto Ducati Luchessi, André Ducati Luchessi*

* Corresponding author
André Ducati Luchessi
E-mail: andre.luchessi@outlook.com

SUPPLEMENTARY FIGURE

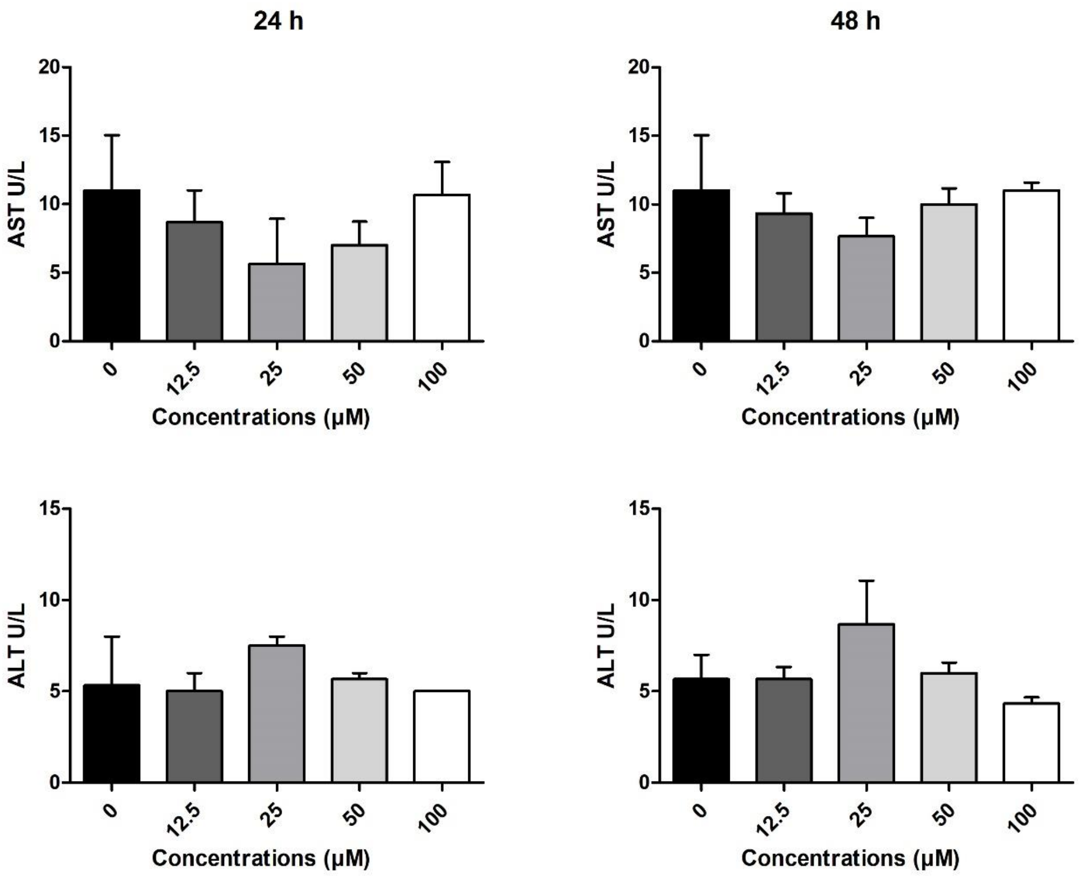

Suppl. Figure 1. AST and ALT values in supernatant of HepG2 cells treated with clopidogrel for 24 and 48 h. Data are shown as mean ± standard error and compared by Kruskal-Wallis.
